# Reducing vertices in property graphs

**DOI:** 10.1371/journal.pone.0191917

**Published:** 2018-02-14

**Authors:** Dominik Tomaszuk, Karol Pąk

**Affiliations:** Institute of Informatics, University of Bialystok, Białystok, Poland; Universidad de las Palmas de Gran Canaria, SPAIN

## Abstract

Graph databases are constantly growing, and, at the same time, some of their data is the same or similar. Our experience with the management of the existing databases, especially the bigger ones, shows that certain vertices are particularly replicated there numerous times. Eliminating repetitive or even very similar data speeds up the access to database resources. We present a modification of this approach, where similarly we group together vertices of identical properties, but then additionally we join together groups of data that are located in distant parts of a graph. The second part of our approach is non-trivial. We show that the search for a partition of a given graph where each member of the partition has only pairwise distant vertices is NP-hard. We indicate a group of heuristics that try to solve our difficult computational problems and then we apply them to check the the effectiveness of our approach.

## 1 Introduction and preliminaries

Graphs are a useful and understandable form of presenting various types of data in areas such as administration, social networks, biological sciences, media, and geography. Property graphs [1] are types of graphs that enable the construction of links, relations, and attributes of particular objects. Some elements of property graphs may be similar and share the same features. These elements can be merged together, and, thanks to that, the property graph becomes smaller, simpler, and easier to process and select the data from.

In this article, we present a way of reducing nodes of a property graph. We rely on the observation that, in the property graph, there may exist elements that have completely different properties, which are non-connectable with each other, and even have disjoint neighbors. The advantage of this approach is that merging different vertex contexts significantly reduces the chance that the query will involve different vertices that have been merged. This approach is subjected to some errors resulting from merging distant vertices and quite random data because of possible relationships. These errors can be eliminated by re-querying only within the merged entities, among which there is very little dependency because vertices are distant in the original graph. Such division speeds up the execution of queries, especially those of high complexity. In our approach to finding this kind of vertex, we use graph vertex coloring methods [2, 3].

Some initial work [4–6] has been done in Resource Description Framework (RDF) [7] and Semantic Web [8], and we are trying to move these ideas to the property graph world. In the article, we also present how to find distant vertices as well as how to find and merge similar ones.

We propose an experimental method of dealing with data similarity problems in property graphs by searching for solutions known from addressing NP-complete graph problems. We also present results obtained with METIS [9] and ColPack [10] support. Our proposals contribute to enable a user who is familiar with graph databases to use and access RDF data and property graph data as well. Property graph databases often have better performance than native RDF graph stores [11–13], so it is important to enable interoperability between these two approaches.

The PG data model rests on the concept of creating directed and key/value-based graphs. It means that there is a tail and head to each edge and both vertices and edges can have properties associated with them.

Following [14, 15], we provide a formal definition below.

**Definition 1** (Property Graph). *A* Property Graph *is a tuple* $PG=\langle V,E,S,P,h_{e},t_{e},l_{v},l_{e},p_{v},p_{e}\rangle$, *where*:

1. *V is a non-empty set of vertices*,
2. *E is a multiset of edges, which are elements of V* × *V*,
3. *S is a non-empty set of character strings*,
4. *P is the Cartesian product S* × *S, where each member has a form p* = 〈*k*, *v*〉 *(property)*,
5. $h_{e}$: *E* → *V is a function that yields the source of each edge (head)*,
6. $t_{e}$: *E* → *V is a function that yields the target of each edge (tail)*,
7. $l_{v}$: *V* → *S is a function mapping each vertex to a label*,
8. $l_{e}$: *E* → *S is a function mapping each edge to a label*,
9. $p_{v}$: *V* → 2^*P*^ *is a function that assigns vertices to their multiple properties, and*
10. $p_{e}$: *E* → 2^*P*^ *is a function that assigns edges to their multiple properties*.

Note that $\langle V,E,h_{e},t_{e},l_{e}\rangle$ is an edge-labeled directed multigraph.

**Fig 1:**
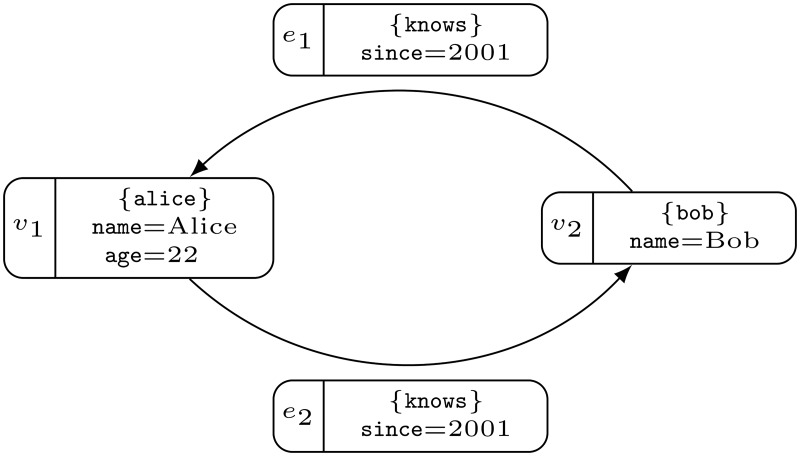
A property graph with two vertices, two edges and four properties. The vertex on the left has an alice label and a property with two key/value pairs: name = Alice, and age = 22, where name and age are keys and Alice and 22 are values. The edge on the top has a knows label and a property since = 2001 key/value pair. The vertex on the right and the edge on the bottom are built similarly.

**Example 1**. *The example in* Fig 1 *presents a property graph*. *This graph includes the following elements*:

$$\begin{array}{c} \begin{array}{ccc} S & = & \{name,Alice,Bob,age,22,since,2001,knows,alice,bob\},\\ V & = & \{v_{1},v_{2}\},\\ p_{v}\left(v_{1}\right) & = & \{\langle name,Alice\rangle ,\langle age,22\rangle \},\\ p_{v}\left(v_{2}\right) & = & \{\langle name,Bob\rangle \},\\ l_{v}\left(v_{1}\right) & = & alice,\\ l_{v}\left(v_{1}\right) & = & bob,\\ E & = & \{e_{1},e_{2}\},\\ h_{e}\left(e_{1}\right) & = & bob,\\ t_{e}\left(e_{1}\right) & = & alice,\\ l_{e}\left(e_{1}\right) & = & knows,\\ p_{e}\left(e_{1}\right) & = & \{\langle since,2001\rangle \},\\ h_{e}\left(e_{2}\right) & = & alice,\\ t_{e}\left(e_{2}\right) & = & bob,\\ l_{e}\left(e_{2}\right) & = & knows,\\ p_{e}\left(e_{2}\right) & = & \{\langle since,2001\rangle \}. \end{array} \end{array}$$

*Note that in* Fig 1 *a property* 〈*name*, *Bob*〉 *is written as name = Bob, respectively*. *Labels* (*i*.*e*. *alice*) *are written in curly braces* (*i*.*e*. *{alice}*). *All property values start with a capital letter*.

On the other hand, RDF constitutes a universal method of the conceptual description or information modeling accessible in Web resources. The elemental constituents of the RDF data model are RDF terms that can be used in reference to resources: anything with identity. The set of RDF terms is divided into three disjoint subsets: IRIs, literals, and blank nodes. Following [7], we provide formal definitions below.

**Definition 2** (IRIs). IRIs *are a set of Unicode names in registered name spaces and addresses referring to registered protocols or name spaces used to identify a resource*.

**Example 2**. <http://dbpedia.org/page/Dog> *is used to identify the dog in DBpedia* [16].

**Definition 3** (Literals). Literals *are a set of lexical forms and datatype IRIs*. *A lexical form is a Unicode string*, *and a datatype IRI is an IRI identifying a datatype*, *where RDF borrows many of the datatypes defined in XML Schema 1.1* [17].

**Example 3**. “*1*” http://www.w3.org/2001/XMLSchema#integer, *where 1 is a lexical form and should be interpreted as integer number*.

**Definition 4** (Blank nodes). Blank nodes *are defined as elements of an infinite set disjoint from IRIs and Literals*.

A collection of RDF triples intrinsically represents a labeled directed multigraph. The nodes are the subjects and objects of their triples. RDF is often referred to as being *graph* where each 〈*s*, *p*, *o*〉 triple can be interpreted as an edge $s\overset{p}{\to }o$. Several of RDF syntax (called *serializactions*) formats exist for writing down graphs. We propose Yet Another RDF Serialization (YARS), which allows prepare RDF data to exchange on the property graph data stores. One example of such a serialization is Yet Another RDF Serialization (YARS) [15], which allow prepare RDF data to exchange on the property graph data stores.

The article is constructed according to sections. In Section 2, we motivate the need for vertex redution in property graphs. Section 3 shows that reducing vertices in property graphs is NP-hard. In Section 4, we introduce tested data sets and our experiments. Section 5 is devoted to related work. The paper ends with conclusions.

## 2 Motivating scenario

Let us suppose we have launched a Web crawler indexing Resource Description Framework in Attributes (RDFa) [18] data, which is a syntax that embeds *RDF triples* [7] in HTML and XML documents. Following [7], we provide definitions of RDF triples below.

**Definition 5** (RDF triple). *Assume that* I *is the set of all Internationalized Resource Identifiers* (*IRIs*), B (*an infinite*) *set of blank nodes*, *and* L *a set of literals*. *An* RDF triple *is defined as a triple t* = 〈*s*, *p*, *o*〉 *where* s∈I⋃B *is called the* subject, p∈I *is called the* predicate, *and* o∈I⋃B⋃L *is called the* object.

**Fig 2:**
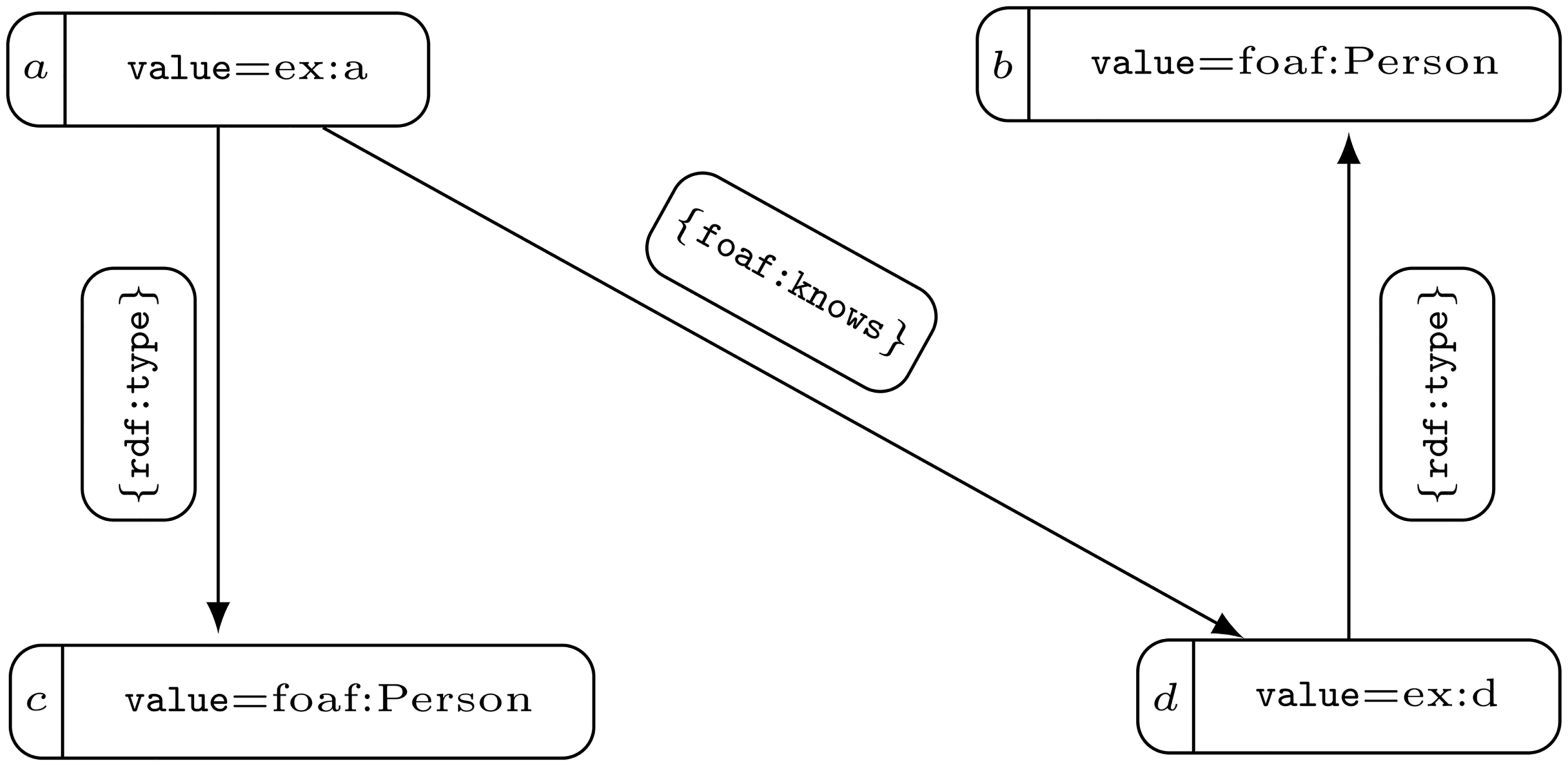
A property graph generated by a web crawler.

Property graph databases are vertex-centric, whereas RDF graph stores are edge-centric. As a result, RDF graph stores use edges, many of which are not critical to our quiries, so we choose property graphs to store our data. The indexed subjects, predicates, and objects of RDF are saved in the graph database in a form of a property graph. Subjects and objects are represented as vertices, whereas predicates are edge labels. These elements can be written in YARS [15], which is a serialization for PG databases that is compatible with RDF. An exemplary property graph can be seen in Fig 2 and Example 4.

**Example 4**. *The example presents YARS*. *Lines 1–2 represent prefix directives*. *Lines 3–5 describe vertex declarations that is a part*, *where vertices are created*. *In lines 6–7 edges and properties are created*. *The example below represents two RDF triples*:

1. :*rdf*: <http://www.w3.org/1999/02/22-rdf-syntax-ns#>
2. :*foaf*: <http://xmlns.com/foaf/0.1/>
3. (*a* {*value*: <http://example.org/p#j>})
4. (*b* {*value*: <http://xmlns.com/foaf/0.1/Person>})
5. (*c* {*value*: “*John Smith*”})
6. (*a*)-[:*rdf*: *type*]->(*b*)
7. (*a*)-[:*foaf*: *name*]->(*c*)

*The first RDF triple consists of* http://example.org/p#j (*an IRI*), http://www.w3.org/1999/02/22-rdf-syntax-ns#type (*an IRI*), *and* http://xmlns.com/foaf/0.1/Person (*an IRI*). *The second RDF triple consists of* http://example.org/p#j (*an IRI*), http://xmlns.com/foaf/0.1/name (*an IRI*), *and John Smith* (*a literal*). *Note that subjects are deduplicated*.

**Fig 3:**
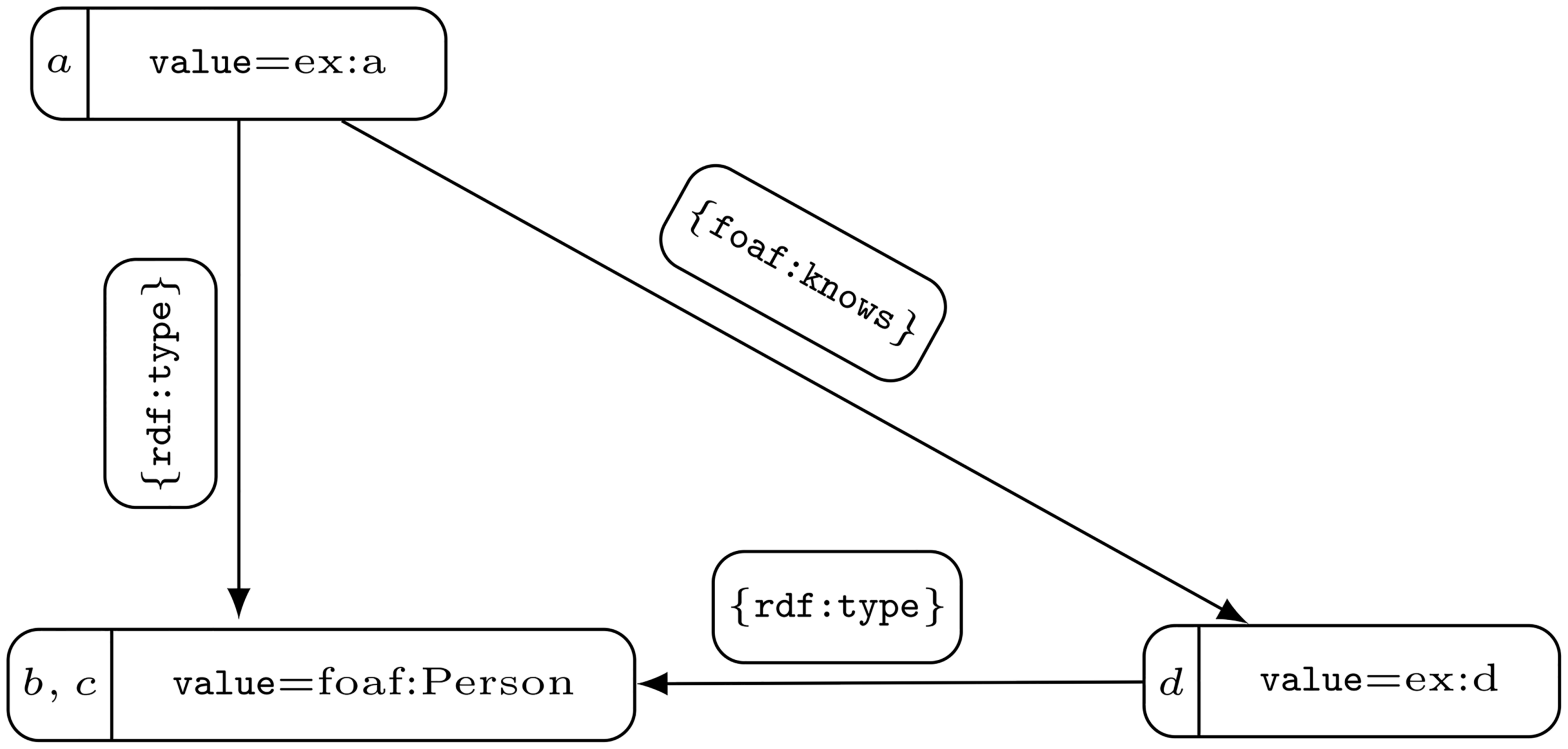
A modification of the graph presented at Fig 2, which is determined by a partition that retains properties as well as has the minimal number of members.

Unfortunately, both subjects and objects may be repeated in different sources that are searched by a Web crawler. For the sake of efficiency, we cannot check with every RDF triple whether nodes of the same name in the database already exist. This is why all subjects and objects of RDF triples encountered in different time intervals are entered into the database. Fig 2 shows repeated vertices and their properties. Such a state of data is not desired, because it causes difficulties with the efficiency of data processing and substantially impedes selecting the data. This is why we would like vertices that represent the same or similar thing to be represented by one node. A way of solving such problems is removing unnecessary nodes with their properties that are the same or similar and merging them into one node. Since a Web crawler indexes RDF triples, we assume that if the vertices have the same properties, they are not connected with the edges (see Fig 3). It is important to note that without this assumption we may encounter a loop in a modified graph, where nodes of the same property have been merged. Indeed, let us consider an edge that connects two vertices of the same property *p* and the vertex *v*_*p*_ that represents the set of all vertices with *p* in the modified graph. Then the edge would be transformed into a loop that links *v*_*p*_ to itself. Such loops are superfluous because of the RDF transformation algorithm to property graphs. Under assumption that there is no edge connecting two vertices with the same property, each set of vertices with a common property is independent. As a result, particular collections of merged nodes of the same property determine the partition of the graph node collection into independent sets. Because, through merging, we wanted to obtain a minimum number of nodes, we assumed that we would also allow the merging of even a few node families, where each would be defined by a common value if no two within the merged family were incidental. Elimination of vertices may take place because the data placed in the properties is similar, hence RDF subjects or objects represent the same family of RDF triple elements. Such minimization leads to a known NP-complete Graph Colorability problem (see GT4 [19]). Note that this problem is originally formulated for undirected graphs, however it is as difficult as the problem of coloring of a directed graph or even a multigraph. Moreover, subfamilies of nodes expressed by the partition of graph into independent sets do not have to be *property closed*. To say that a partition is property closed means simply that every two vertices that have the same property have to belong to the same independent sets. This property does not have to be kept even in the case of a graph of several vertices. Indeed, the graph presented in Fig 2 is 2-colorable, and such coloring is only determined by {{*a*, *b*}, {*c*, *d*}} partition. However, this partition is not closed to the Person property (see nodes *b*, *c*). It is easily noticeable that only coloring, that retains the property, including Person, is determined by {{*a*}, {*b*, *c*}, {*d*}} partition (see Fig 3).

Therefore, we can not directly use any approximation algorithm for graph coloring to reduce the number of vertices in a property graph. To solve this problem in our approach, first we transform a given property graph, sticking together vertices that have the same property as at Fig 3. Then we color a graph determined by classes of properties, and finally we assign to each vertex the color of the class to which it belongs. We describe the transformation more formally in Section 3 and use it to show in Theorem 1 that our reduction problem is NP-hard. Additionally, in Theorem 2 we show that based on this approach, we can obtain a reduced property graph that has the minimal number of vertices.

## 3 Reducing vertices

In this section, we will show that even in the case of narrowing the scope of searching for minimal colorings to divisions into independents sets that are closed because of a common property, the problem of minimal coloring is also an NP-hard. To do this, we will first enter the necessary definitions and notations so as to prove that our problem is NP-hard. Subsequently, we will suggest a method enabling the use of known approximating algorithms in search for minimal graph coloring. For this purpose, we will present a method of converting a property graph into an undirected graph whose minimal coloring will unequivocally designate minimal property graph coloring.

To formulate our coloring problem, we need to set the appropriate vocabulary of notations. Let G=〈V,E〉 be a simple undirected graph with the vertex set V and the edge set E, where each edge is a two-element subset of V. We assume that considered undirected graphs are loopless since a vertex that is incident to a loop could never be properly colored. A subset $V_{1}$ of V is called independent if {v,u}∉E for all $v,u\in V_{1}$. For simplicity, the phrase *π* is a partition of G means that *π* is a partition of V into mutually disjoint sets.

Now we can formulate the Graph Colorability problem as the following decision problem:

**Graph Colorability** (**GT4**):

 Instance: An undirected graph G=〈V,E〉, a positive integer k≤|V|.

 Question: Does a partition of G into *k* independent sets exist?

Let $PG=\langle V,E,S,P,h_{e},t_{e},l_{e},p_{v},p_{e}\rangle$ be a property graph. We call *PG single*, if each vertex has exactly one property, i.e., for every *v* ∈ *V* there exits *p* ∈ *P* such that $p_{v}(v)=\{p\}$. We call *PG unique adjacency* if the adjacent vertices have no common property, i.e., $p_{v}(h_{e}(a))\cap p_{v}(t_{e}(a))=\emptyset$ for every *a* ∈ *E*. Note that if *PG* is single then the *unique adjacency* property can be expressed as follows: $p_{v}(h_{e}(a))\neq p_{v}(t_{e}(a))$ for every *e* ∈ *E*. Let *V*_1_ be a subset of *V*. We call *V*_1_ *independent* if $h_{e}(a)\notin V_{1}$ or $t_{e}(a)\notin V_{1}$ for all *a* ∈ *E*. Let *p* be a property. We call *V*_1_ the *class* of *p* if each vertex of *V*_1_ has property *p* and each vertex that has property *p* belongs to *V*_1_, i.e., $u\in V_{1}\Leftrightarrow p\in p_{v}(u)$ for every *u* ∈ *V*. Finally, we call *V*_1_ *property closed* if for every *p* ∈ *P* whose the class has a non-empty intersection with *V*_1_ and holds the class of *p* is a subset of *V*_1_.

**Combined Property Graph** (**CPG**):

 Instance: A single property graph $PG=\langle V,E,S,P,h_{e},t_{e},l_{v},l_{e},p_{v},p_{e}\rangle$, a positive integer *k* ≤ |*V*|.

 Question: Does a partition of *V* into *k* independent property closed sets in *PG* exist?

It is important to note that there exists a partition of *V* into mutually disjoint independent property closed sets since the property graph *PG* is single. We show that a non-single property graph may not have such a partition. Let us consider a property graph presented at Fig 2 that contains vertices *a*, *b*, *c*. Denote by *V*_*b*_ an arbitrary property closed subset of vertices that contains *b*. Suppose that *b* has an additional property: value = ex:a (value is a key, and ex:a is a value of property). As *a* and *b* have the property value = ex:a we infer that *a* ∈ *V*_*b*_. Similarly, *c* ∈ *V*_*b*_ since value = foaf:Person is a property of *b* and *c*. But vertices *a* and *c* are connected by an edge. This contradicts our assumption that *V*_*b*_ is independent.

We can also consider weakened assumption that *PG* is single, preserving the existence of such a partition. Indeed, we can consider a family of property graphs where, if each pair of property closed subsets of vertices has a common member in their class, the class determined by one of them is a subset of the class for the second one. However, we can easily transform such property graphs to single ones, removing every property where the determined class is a subset of the class of another property.

**Theorem 1**. **CPG** *is NP-hard*.

*Proof*. We transform Graph Colorability to **CPG**. Let G=〈V,E〉 be an undirected graph and a positive integer k≤|V|. Without loss of generality we can assume that V={1,2,…,n} where n=|V| (note that we can consider an injection function ϕ:V↦{1,2,…,|V|} that assigns a number to each vertex of a graph).

**Fig 4:**
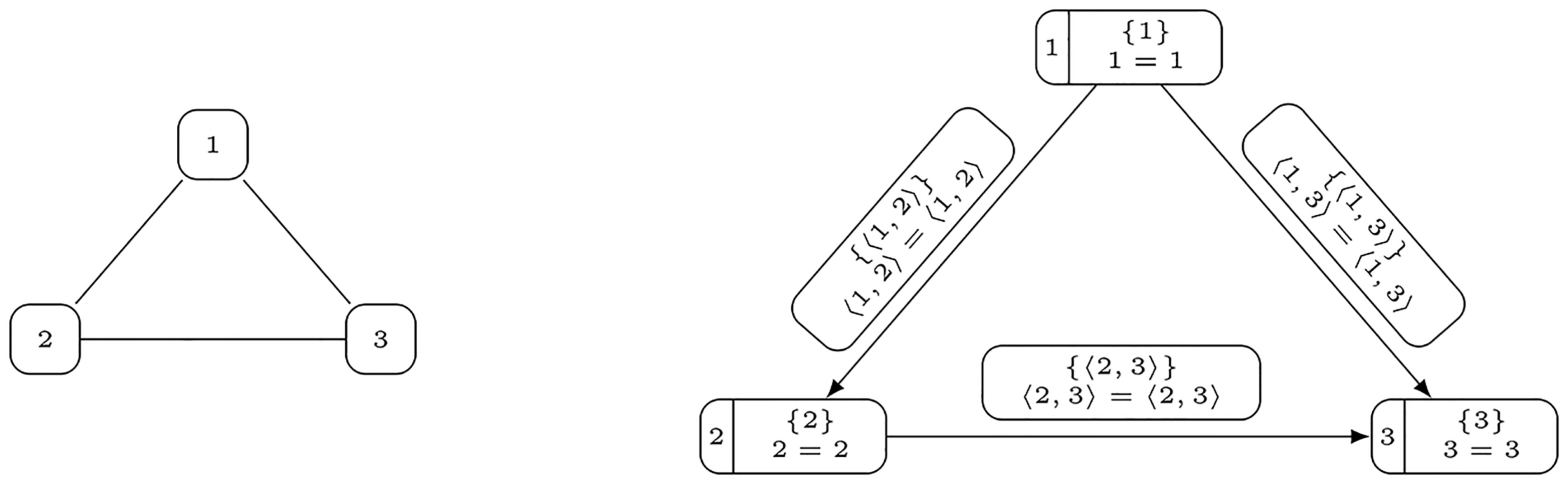
An illustration of the transformation construction from the proof of Theorem 1 that gives the property graph on the right hand side for an undirected graph presented on the left hand side.

We describe a property graph as a tuple $PG=\langle V,E,S,P,h_{e},t_{e},l_{v},l_{e},p_{v},p_{e}\rangle$ defined by

$$\begin{array}{c} \begin{array}{ccc} V & = & V,\\ E & = & \{\langle v,u\rangle :\{v,u\}\in E\wedge v<u\},\\ S & = & V⋃E,\\ P & = & \{\langle p,q\rangle :p,q\in S\},\\ h_{e}\left(e\right) & = & u\;\text{if}\;e=\langle v,u\rangle ,\\ t_{e}\left(e\right) & = & v\;\text{if}\;e=\langle v,u\rangle ,\\ l_{v}\left(v\right) & = & v,\\ l_{e}\left(e\right) & = & e,\\ p_{v}\left(v\right) & = & \{\langle v,v\rangle \},\\ p_{e}\left(e\right) & = & \{\langle e,e\rangle \}, \end{array} \end{array}$$

where *v*, *u* ∈ *V* and *e* ∈ *E* (see Fig 4). Note that this translation can clearly be done in LOGSPACE.

The main idea of proofs uses the fact that for every property there exists at most one vertex or one edge that has the property or more precisely

$$\begin{array}{c} \forall_{p\in P}\;(|\left\{v\in V:p_{v}\left(v\right)=p\right\}\left|\leq 1\wedge \right|\left\{e\in E:p_{v}\left(e\right)=p\right\}|\leq 1). \end{array}$$ (1)

Indeed, each property *p* that is assigned to a vertex or an edge has the following form 〈*x*, *x*〉, where x∈V⋃E. Additionally, V and E do not have common members, since each edge is a two-element subset of natural numbers and each vertex is a natural number. Based on this observation we show that there exists a partition of G into *k* independent sets if and only if there exists a partition of *PG* into *k* independent property closed sets.

Let us consider a partition *π* of G into *k* independent sets. Consider *π*′ = *π* as a partition of *PG* since (V=V). We show that *π*′ is a partition of *PG* into *k* independent property closed sets. To do this, first, we show that each member of *π*′ is an independent set of *PG*. Let us consider an arrow *a* ∈ *E* and suppose contrary that exists *V*_1_ ∈ *π*′ such that $h_{e}(a)\in V_{1}\wedge t_{e}(a)\in V_{1}$. The arrow *a* can be represented in the form 〈*v*, *u*〉 for some *v*, *u* ∈ *V* where {v,u}∈E and *v* < *u*. Then $h_{e}(a)=u,t_{e}(a)=v$, so imply that {*v*, *u*} is an edge that connects two members of *V*_1_, but *V*_1_ is an independent set of G as a member of *π*, contradiction. We are now in a position to show that each member of *π*′ is a property closed set. But the justification of the property is obvious, since there is no two vertices that have the same property by (1), and the proof of the first implication is complete.

Let us consider a partition *π*_*PG*_ of *PG* into *k* independent property closed sets. Similarly, let us consider $\pi_{PG}^{\prime }=\pi_{PG}$ as a partition of G. Suppose, contrary to our claim that $\pi_{PG}^{\prime }$ is not a partition of G into independent sets. Then there exists $V_{2}\in \pi_{PG}^{\prime }$ and two vertices *v*, *u* ∈ *V*_2_ such that {v,u}∈E. Without loss of generality we can assume that *v* < *u*. Then 〈*v*, *u*〉 ∈ *E* connects two members of *V*_2_ being an independent set of *PG*, since $h_{e}\left(\langle v,u\rangle \right)=u,t_{e}\left(\langle v,u\rangle \right)=v$, and the proof is complete.

Let $PG=\langle V,E,S,P,h_{e},t_{e},l_{v},l_{e},p_{v},p_{e}\rangle$ be a property graph. Let *π*_*PG*_ be a set of non-empty subsets of *V* such that *V*′ is a member of *π*_*PG*_ if and only if there exists a property *p* ∈ *P* for which *V*′ is the set of all vertices of *V* that have property *p*. Obviously, if *PG* is single, then *π*_*PG*_ is a partition of *PG*. Moreover, if *PG* is unique adjacency then, by definition, there are no two vertices that are connected by an arrow and belong to the same member of *π*_*PG*_. Hence, each member of *π*_*PG*_ is an independent set for every unique adjacency property graph *PG*. For subsets *V*_1_, *V*_2_ of *V* we use the following notation *V*_1_∼_*PG*_ *V*_2_ if there exists an edge *e* ∈ *E* that connects a member of *V*_1_ with a member of *V*_2_ or more precisely

$$\begin{array}{c} V_{1}{∼}_{PG}V_{2}\Leftrightarrow \exists_{e\in E}\left(h_{e}\left(e\right)\in V_{1}\wedge t_{e}\left(e\right)\in V_{2}\right)\vee \left(h_{e}\left(e\right)\in V_{2}\wedge t_{e}\left(e\right)\in V_{1}\right). \end{array}$$ (2)

Now, we define a partition graph G(PG,π) for an arbitrary partition *π* of *V* as follows. The set of vertices in G(PG,π) equals *π* and two vertices *V*_1_, *V*_2_ of G(PG,π) are connected if *V*_1_ ≠ *V*_2_ and *V*_1_∼_*PG*_ *V*_2_.

Let us consider a partition *σ* of the set of vertices in G(PG,π) and a member *V*′ ∈ *σ*. Note that ⋃_*x* ∈ *V*′_ *x* is a subset of *V*, since each member of *σ* is a set of nonempty subsets of *V*. In consequence,

$$\begin{array}{c} P^{V}\left(\sigma \right)=\left\{\underset{x\in V^{\prime }}{⋃}x:V^{\prime }\in \sigma \right\} \end{array}$$ (3)

is a set of nonempty subsets of *V*. Note that $P^{V}\left(\sigma \right)$ is also a partition of G. Indeed, $P^{V}\left(\sigma \right)$ covers the whole *V* since, for each vertex *v* ∈ *V* there exist *V*′ ∈ *π*, *V*″ ∈ *σ* such that *v* ∈ *V*′ ∈ *V*″, hence *v* ∈ ⋃_*x* ∈ *V*″_ *x*. Additionally, if two members of $P^{V}\left(\sigma \right)$, e.g., ${⋃}_{x\in V_{1}^{\prime \prime }}x,{⋃}_{x\in V_{2}^{\prime \prime }}x$, have a common vertex *v*, then there exist $V_{1}^{\prime }\in V_{1}^{\prime \prime }$, $V_{2}^{\prime }\in V_{2}^{\prime \prime }$ such that $V_{1}^{\prime }∍v\in V_{2}^{\prime }$. But *π*, *σ* have mutually disjoint members, hence $V_{1}^{\prime }=V_{2}^{\prime }$ and $V_{1}^{\prime \prime }=V_{2}^{\prime \prime }$, and finally these two members of $P^{V}\left(\sigma \right)$ are equals.

Similarly, if *π* is property closed then each member *V*′ of *π* that has a common element with a member *V*″ of *π*_*PG*_, has to contain whole *V*″, i.e., *V*″ ⊆ *V*′. In consequence, we can assign a partition of $G(PG,\pi_{PG})$ to the partition *π*. This partition is defined as follows:

$$\begin{array}{c} a\in P^{\pi_{PG}}\left(\pi \right)\Leftrightarrow \exists_{V^{\prime }\in \pi }\;a=\left\{V^{\prime \prime }\in \pi_{PG}:V^{\prime \prime }\subseteq V^{\prime }\right\}. \end{array}$$ (4)

We show that based on $G(PG,\pi_{PG})$ we can adapt each known approximation algorithm for graph coloring to **CPG** problem.

**Theorem 2**. *Let PG be single unique adjacency property graph*. *Then*

(i) *for every π be a partition of PG into k independent property closed sets holds* $P^{\pi_{PG}}\left(\pi \right)$ *is a partition of* $G(PG,\pi_{PG})$ *into k independent sets*, *and*
(ii) *for every π be a partition of* $G(PG,\pi_{PG})$ *into k independent sets holds* $P^{V}\left(\pi \right)$ *is a partition of PG into k-independent property closed sets*.

*Proof*.

(i) Let *π* be a partition of *PG* into *k* independent property closed sets. Obviously, $P^{\pi_{PG}}\left(\pi \right)$ is a partition of $G(PG,\pi_{PG})$ and has *k* members, which is clear from (4). We showed that each member of $P^{\pi_{PG}}\left(\pi \right)$ is an independent set. Let us consider $a\in P^{\pi_{PG}}\left(\pi \right)$ and suppose contrary to our claim, that there exist *x*, *y* ∈ *a* for which {*x*, *y*} is an edge of $G(PG,\pi_{PG})$. From (2), there exists *e* ∈ *E* such that $h_{e}\left(e\right)\in x\wedge t_{e}\left(e\right)\in y$ or $h_{e}\left(e\right)\in y\wedge t_{e}\left(e\right)\in x$. Without loss of generality we can assume that $h_{e}\left(e\right)\in x\wedge t_{e}\left(e\right)\in y$. Additionally, from (4), there exists *V*′ ∈ *π* satisfying *a* = {*V*′ ∈ *π*_*PG*_: *V*″ ⊆ *V*′}. Hence *x*, *y* ⊆ *V*′, and finally $h_{e}\left(e\right),t_{e}\left(e\right)\in V^{\prime }$. This contradicts the fact that *V*′ as a member of *π* is independent.
(ii) Let *π* be a partition of $G(PG,\pi_{PG})$ into *k* independent sets. It is evident that $P^{V}\left(\pi \right)$ is a partition of *PG* and has *k* members. Let us consider a member *a* of $P^{V}\left(\pi \right)$. Then there exist *V*′ ∈ *π* satisfying *a* = ⋃_*x* ∈ *V*′_ *x*.
  We show first that *a* is an independent property closed. Suppose first that *a* is not independent. Then there exists an edge *e* ∈ *E* such that $h_{e}\left(e\right)\in a\wedge t_{e}\left(e\right)\in a$, and in consequence there exist *x*_1_, *x*_2_ ∈ *V*′ with the following properties: $h_{e}\left(e\right)\in x_{1}$ and $t_{e}\left(e\right)\in x_{2}$. But then from (2), we obtain that {*x*_1_, *x*_2_} is an edge of G(PG,π), and in consequence *V*′ contains two connected element *x*_1_, *x*_2_. This contradicts the fact that *V*′ as a member of *π* is an independent set.
  Now we prove that *a* is property closed. Let us consider *q* ∈ *P* whose class has a non-empty intersection with *a*. To be specific there exists *u* ∈ *a* with ${qinp}_{v}(u)$. Then there exists *x*_1_ ∈ *V*′ such that *u* ∈ *x*_1_. Moreover, *x*_1_ ⊆ (⋃_*x*∈*V*′_ *x*) = *a*, and *x*_1_ as a member of *V*′ is an element of *π*_*PG*_, hence *x*_1_ is a class of a property *q*_1_ ∈ *P* and *q*_1_ ∈ $p_{v}(u)$. But since *PG* is single, *u* has exactly one property, therefore *q* = *q*_1_ and in consequence the class of *q*, which is equal to *x*_1_, is a subset of *a*. This concludes the proof.

## 4 Experiments and evaluation

In this section, we will present a description of data sets and experiments from three areas. The first group of experiments (Subsection 4.2) shows property graph coloring characteristics. In Subsection 4.3, we show how our proposal works on graph databases. The last group of experiments (Subsection 4.4) presents how property graph serializations deal with our solutions.

### 4.1 Data sets description and set-up

All experiments were executed on an Intel Core i7-4770K CPU @ 3.50GHz (4 cores, 8 threads), 8GB of RAM (clock speed: 1600 MHz), and an HDD with reading speed rated at ∼160 MB/sec (we test it in hdparm -t). We used Linux Mint 17.3 Rosa (kernel version 3.13.0), Python 3.4.3 with RDFLib 4.2.1, gcc 4.8.4, and Docker 1.12.3.

We gathered data sets from the Web in five ways: crawled data via modified LDSpider [20], subset of DBpedia [21], subset of Wikidata [22], W3C Public Mailing List Archives (https://lists.w3.org/) and automatically generated using Berlin SPARQL Benchmark (BSBM) [23].

The first data set (ds_1_) was generated in LDSpider, which was extended with YARS support. The data set mainly concerns Friend of a Friend (FOAF) [24] information because we used FOAF URIs in the seed file. FOAF is a vocabulary that describes persons, their activities and relations with other people and objects. The next data set (ds_2_) was created on the basis of DBpedia 3.0 [21], which contains data from different infoboxes in Polish. The third data set (ds_3_) is a dump (access date: 2016-06-21) at the class hierarchy of Wikidata Properties [22], which is structured data of its Wikimedia sister projects including Wikipedia, Wikisource, and others. The fourth data set (ds_4_) was found on the W3C Public Mailing List (https://goo.gl/9x2WAu). This data set was generated in cwm.py (that is a data processor and reasoner for the Semantic Web and has data about class/property equivalences and other Web Ontology Language [25] metadata. The last data set (ds_5_) is generated using Berlin SPARQL Benchmark (BSBM) [23], which was extended with YARS support. Description of data sets is presented in Table 1.

**Table 1:** Description of data sets.

Characteristics	ds_1_	ds_2_	ds_3_	ds_4_	ds_5_
RDF triples	n/a	27063	2691	5506	n/a
File sizes					
*S*_*nt*_ [*B*]	n/a	3780046	362172	713761	n/a
$S_{yars}^{b}$ [*B*]	3776452	8180057	816951	1523455	9084098
$S_{yars}^{a}$ [*B*]	2386717	4865661	564074	786257	4940874
RDF →YARS [*s*]	n/a	4.318	0.370	0.629	n/a
*R*_*d*_ [%]	63.20	59.48	69.05	51.61	54.39
Property graph entities					
Vertices	23690	54124	5382	10228	45252
Unique vertices	9611	16777	2519	1142	8623
Edges	10876	26649	2691	3970	21104

ds_2_, ds_3_ and ds_4_ are serialized RDF, so we had to transform thom into YARS, which is indirect PG serializaction for RDF data. Our transformation tool was built in Python, and it is available at https://github.com/domel/yars. All of the considered data sets use YARS so that they can be compared under the same conditions. Description of data sets is presented in Table 1. The first part of the table shows input files characteristics such as N-Triples size ($S_{yars}^{b}$), YARS size before removing the repeated nodes ($S_{yars}^{b}$), YARS size after removing the repeated nodes ($S_{yars}^{a}$). Generation times are presented in the second part of the table. The third part presents reducing duplicates ratio (*R*_*d*_) of $S_{yars}^{a}$ to $S_{yars}^{b}$. The last part of the table shows graph characteristics.

### 4.2 Property graph coloring

Our tool for property graph coloring uses ColPack [10] and METIS [9] file format. Our tool for METIS and other graph formats was built in C++ and it is available at https://github.com/domel/graph_syntax. Our main tool for PG coloring is available at https://github.com/domel/pg_color.

To reduce vertices in property graphs we have to take five steps:

1. remove deduplicate vertices,
2. transform YARS into METIS,
3. color graph,
4. reduce vertices,
5. transform METIS into YARS.

In Table 2, we present transformation from YARS into METIS and its characteristics. The first part of the table shows input files characteristics such as YARS size before removing the repeated nodes ($S_{yars}^{b}$), YARS size after removing the repeated nodes ($S_{yars}^{a}$), and METIS size (*S*_*metis*_). In the second part of the table, we present the arithmetic mean time of transformation from 10 runs. The results show that the regularity of the graph has a strong influence on the transformation time, i.e., ds_2_ is irregular in its structure and its transformation time is worse than ds_5_, which is benchmark generated. The third part of the table shows output file characteristics such as YARS with color metadata size (${}_{o}S_{yars}^{cm}$), regular YARS file size (_*o*_ *S*_*yars*_), and Lzip/DEFLATE [26], tANS (ZStandard implementation: http://facebook.github.io/zstd/) [27] and Brotli [28] stream compressed YARS (${}_{o}S_{yars}^{lzip}$, ${}_{o}S_{yars}^{tans}$ and ${}_{o}S_{yars}^{br}$). The last part shows reducing ratios referring to YARS with color metadata (*R*^*c*^) and YARS after removing the repeated nodes (*R*^*a*^). In addition, we show the ratio of YARS before removing the repeated nodes (*R*^*b*^) and YARS after removing the repeated nodes with compression ($R_{lzip}^{a}$, $R_{tans}^{a}$ and $R_{br}^{a}$). Database servers [29–31] often can send messages to clients via HTTP [32] or RESTful web services [33]. Therefore, we decided to test our solution for compression to improve transfer speed and bandwidth utilization.

**Table 2:** Summary of experiments.

Characteristics	ds_1_	ds_2_	ds_3_	ds_4_	ds_5_
File sizes					
$S_{yars}^{b}$ [*B*]	3776452	8180057	816951	1523455	9084098
$S_{yars}^{a}$ [*B*]	2386717	4865661	564074	786257	4940874
*S*_*metis*_ [*B*]	95137	245120	18005	29108	180054
Times					
YARS → METIS [*s*]	3.548	13.188	0.272	0.106	3.574
Coloring time [*s*]	0.092	2.906	0.365	0.073	0.612
Reducing time [*s*]	2.728	9.611	0.364	0.129	2.915
Total time [*s*]	6.368	25.705	1.001	0.308	7.101
File sizes					
${}_{o}S_{yars}^{cm}$ [*B*]	2793367	5734691	669874	906578	5555733
_*o*_ *S*_*yars*_ [*B*]	1390302	2826548	361010	452664	3439329
${}_{o}S_{yars}^{lzip}$ [*B*]	170443	375414	24014	48676	709976
${}_{o}S_{yars}^{tans}$ [*B*]	141914	312572	19971	40552	591215
${}_{o}S_{yars}^{br}$ [*B*]	116613	261997	14403	36862	537261
Reducing ratios					
*R*^*c*^ [%]	73.97	70.11	82.00	59.51	61.16
*R*^*a*^ [%]	58.25	58.09	64.00	57.57	69.61
*R*^*b*^ [%]	36.82	34.55	44.19	29.71	37.86
$R_{lzip}^{a}$ [%]	4.51	4.59	2.94	3.20	7.82
$R_{tans}^{a}$ [%]	3.76	3.82	2.44	2.66	6.51
$R_{br}^{a}$ [%]	3.09	3.20	1.76	2.42	5.91

To test our approach we have to choose the minimum distance that has to be maintained between each pair of vertices merged together. At the same time, we have to choose the minimum distance between vertices of the same color. Below we present two cases: distance-1 and distance-2 [2].

**Fig 5:**
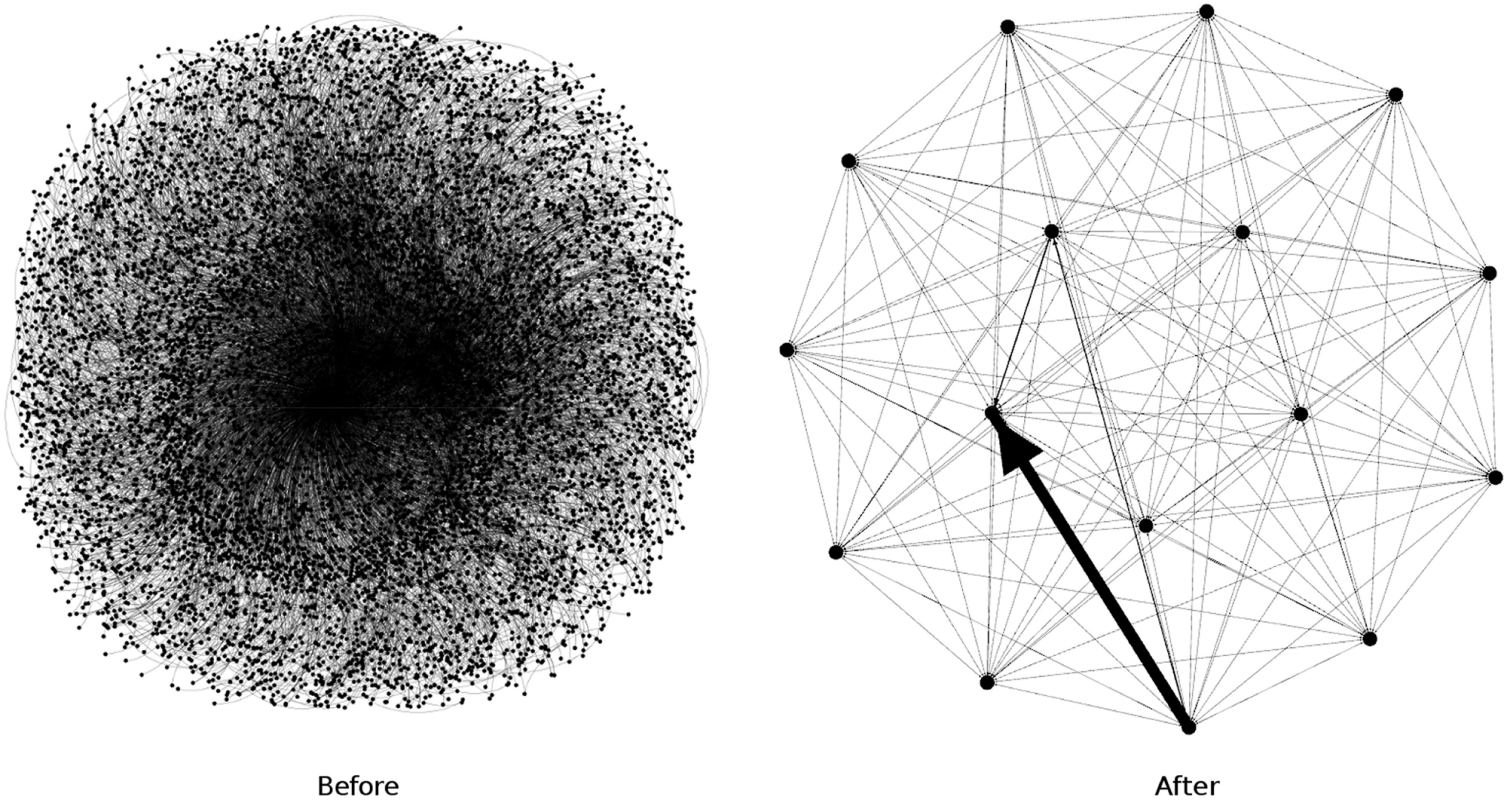
Distance-1 coloring of DS_1_.

**Fig 6:**
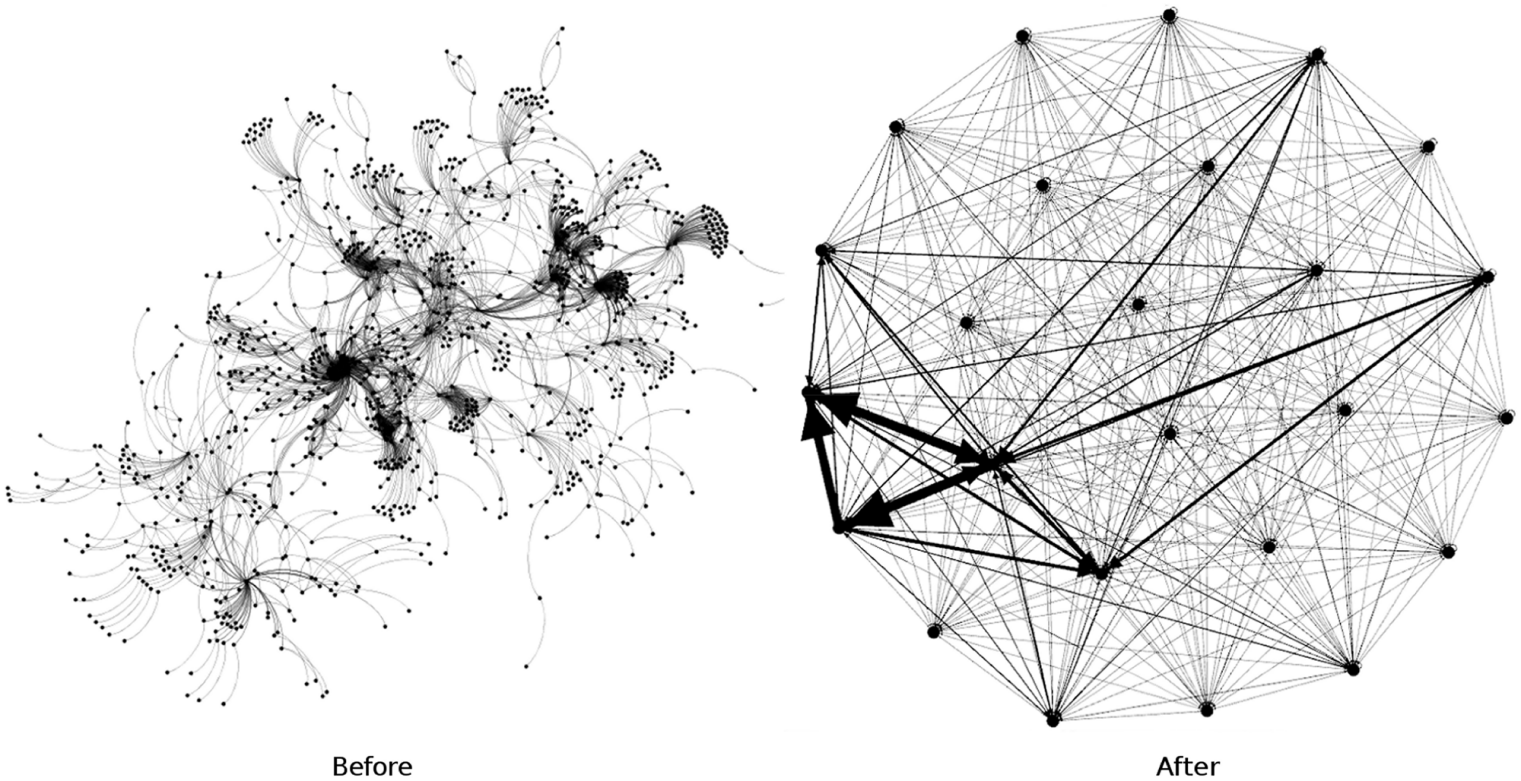
Distance-1 coloring of DS_4_.

In Table 3, we present distance-1 coloring characteristics, such as unique vertex reducing ratio (*R*^*u*^) of unique vertices to output vertices, total vertex reducing ratio (*R*^*t*^) of input vertices to output vertices. Figs 5 and 6 present ds_1_ and ds_4_ before and after our reducing vertices. To visualize these graphs we use the Yifan Hu algorithm (before) [34] and the Fruchterman-Reingold algorithm (after) [35].

**Table 3:** Distance-1 coloring.

Characteristics	ds_1_	ds_2_	ds_3_	ds_4_	ds_5_
Vertices (input)	23690	54124	5382	10228	45252
Unique vertices (input)	9611	16777	2519	1142	8623
Vertices (output)	15	5	4	24	4
Vertex reducing ratios					
*R*^*u*^ [%]	0.16	0.03	0.16	2.10	0.05
*R*^*t*^ [%]	0.06	0.01	0.07	0.23	0.01

**Fig 7:**
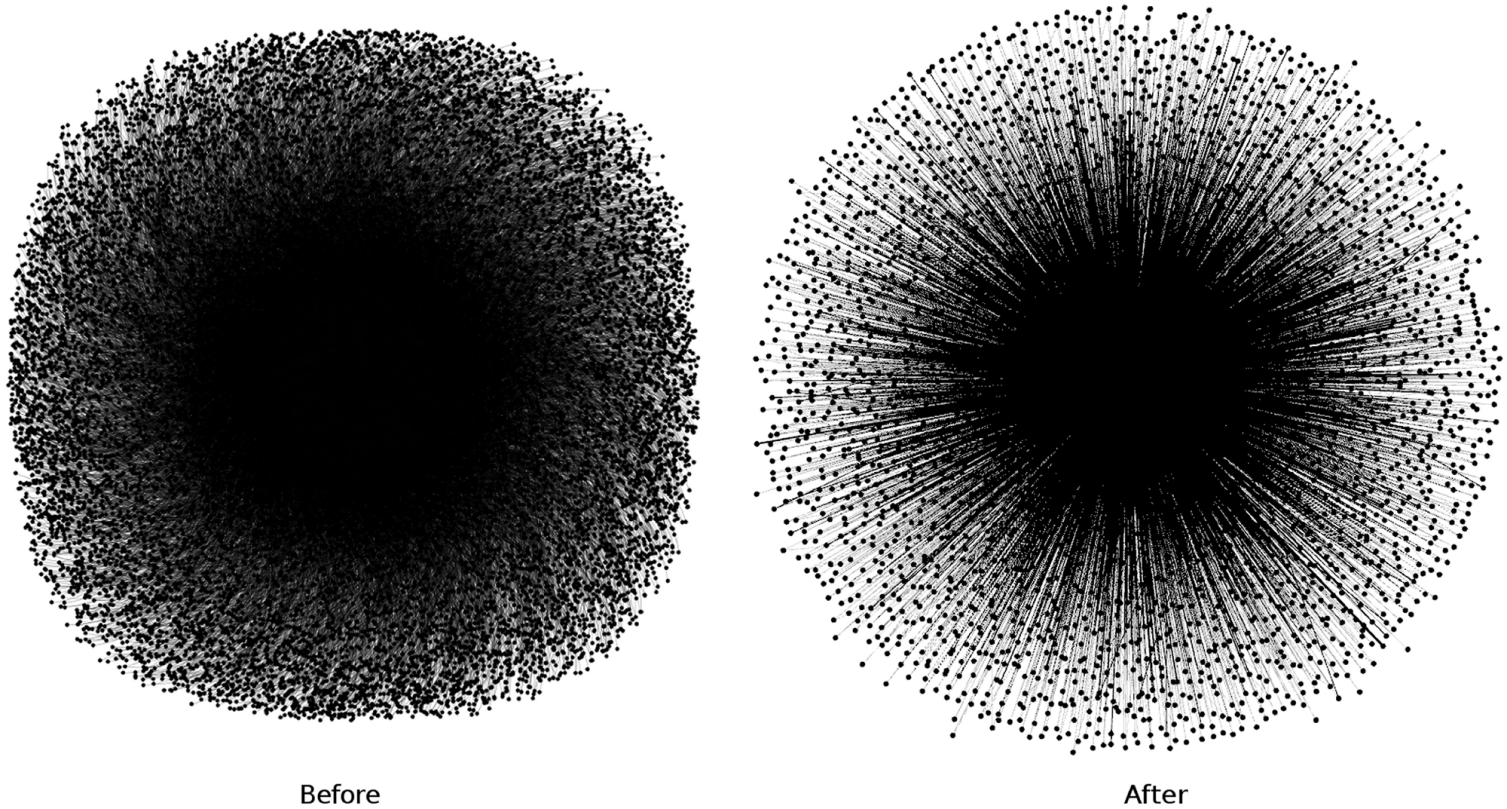
Distance-2 coloring of DS_2_.

**Fig 8:**
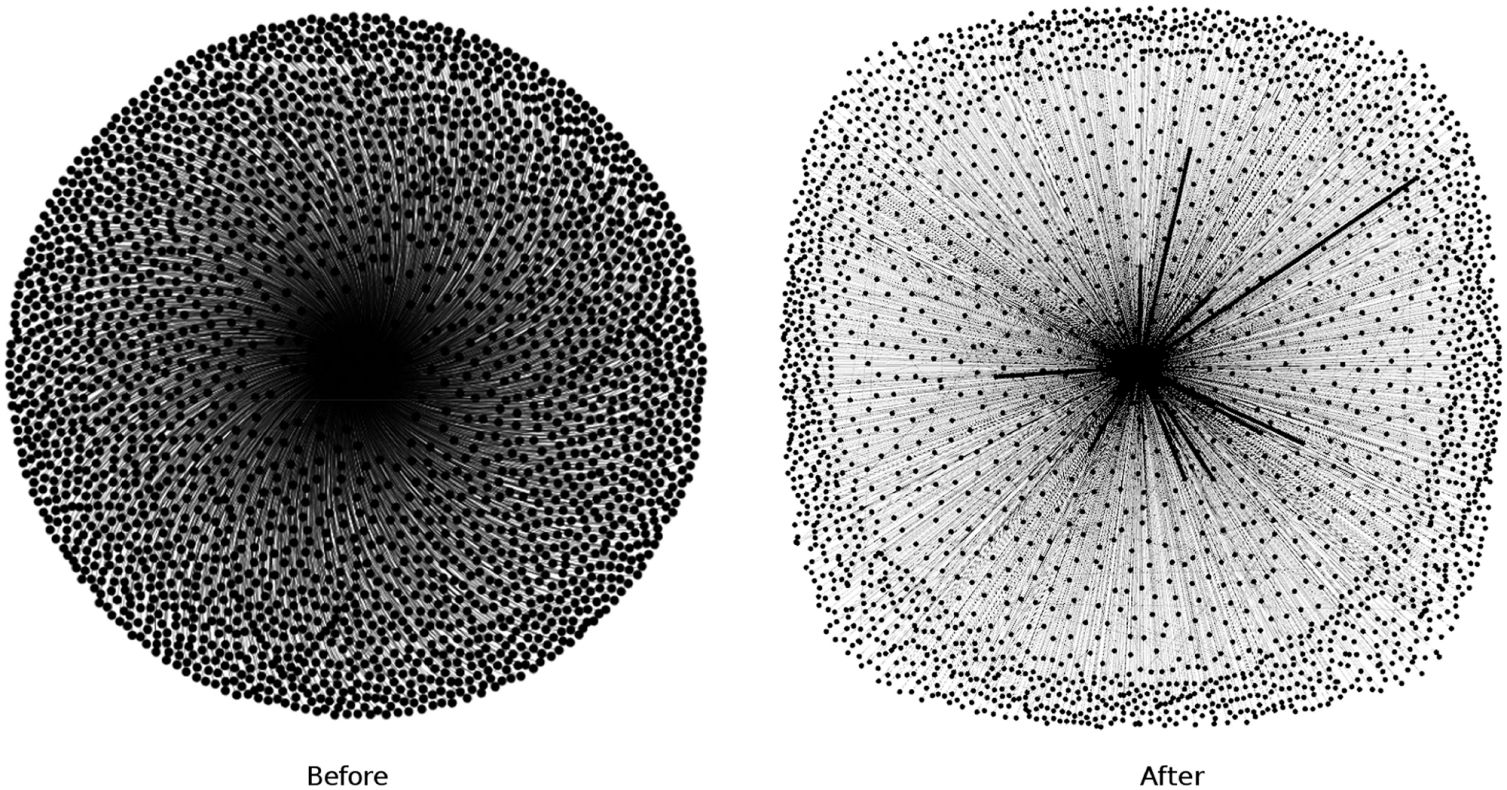
Distance-2 coloring of DS_3_.

In Table 4, we present distance-2 coloring characteristics, such as unique vertex reducing ratio (*R*^*u*^), total vertex reducing ratio (*R*^*t*^), which show, how many nodes of the same color are removed. Figs 7 and 8 present ds_2_ and ds_3_ before and after us reducing vertices. To visualize these graphs we use the Yifan Hu algorithm.

**Table 4:** Distance-2 coloring.

Characteristics	ds_1_	ds_2_	ds_3_	ds_4_	ds_5_
Vertices (input)	23690	54124	5382	10228	45252
Unique vertices (input)	9611	16777	2519	1142	8623
Vertices (output)	2036	2680	2486	127	1285
Vertex reducing ratios					
*R*^*u*^ [%]	21.18	15.97	98.69	11.12	14.9
*R*^*t*^ [%]	8.54	4.95	46.19	1.24	2.84

### 4.3 Querying graph databases

In this subsection, we show our experiments considering querying ds_1_, ds_2_, ds_3_, ds_4_ and ds_5_ before and after us reducing vertices in common graph databases, such as Neo4j [30], Titan 1.0.0 [29], and OrientDB 2.2.14 [31]. All these property graph databases were running in Docker [36], which is a completely sandboxed virtual environment. The instructions to build images (i.e. *dockerfiles* are avaliable on:

1. Neo4j 3.0.1: https://hub.docker.com/_/neo4j/,
2. Titan 1.0.0: https://hub.docker.com/r/elubow/titan-gremlin/,
3. OrientDB 2.2.14 https://hub.docker.com/_/orientdb/.

**Fig 9:**
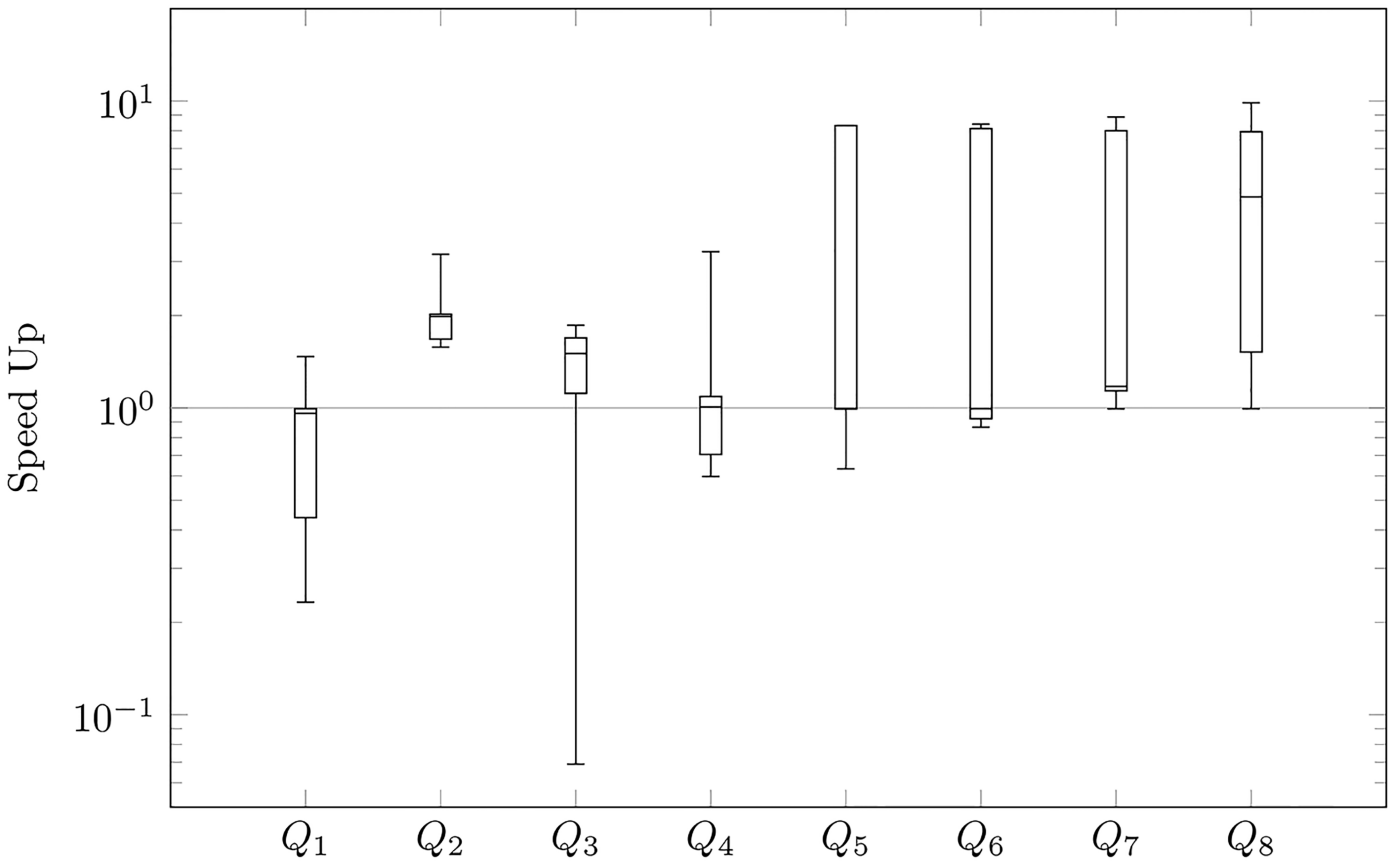
Speed-up of Neo4j querying.

**Fig 10:**
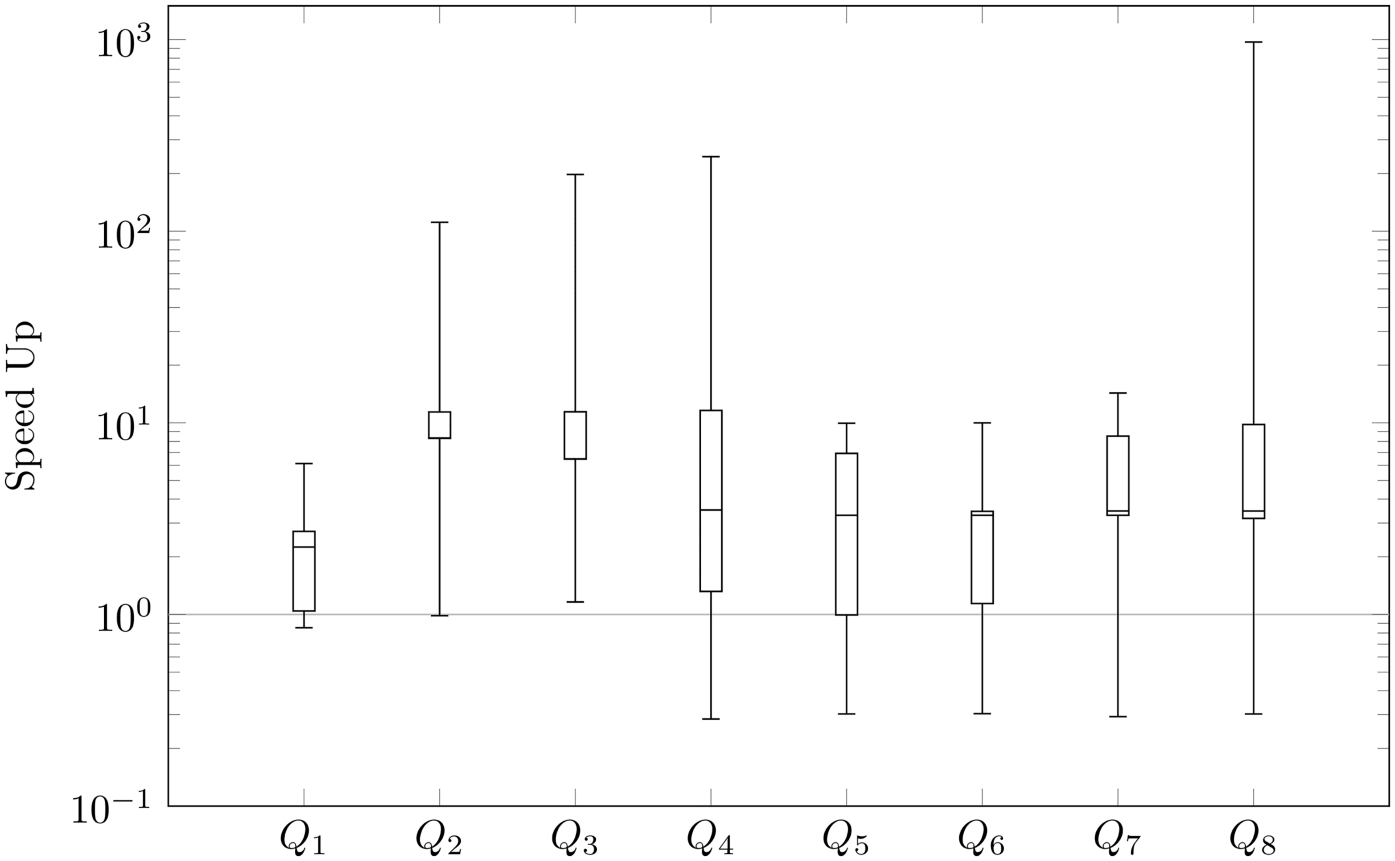
Speed-up of Titan querying.

**Fig 11:**
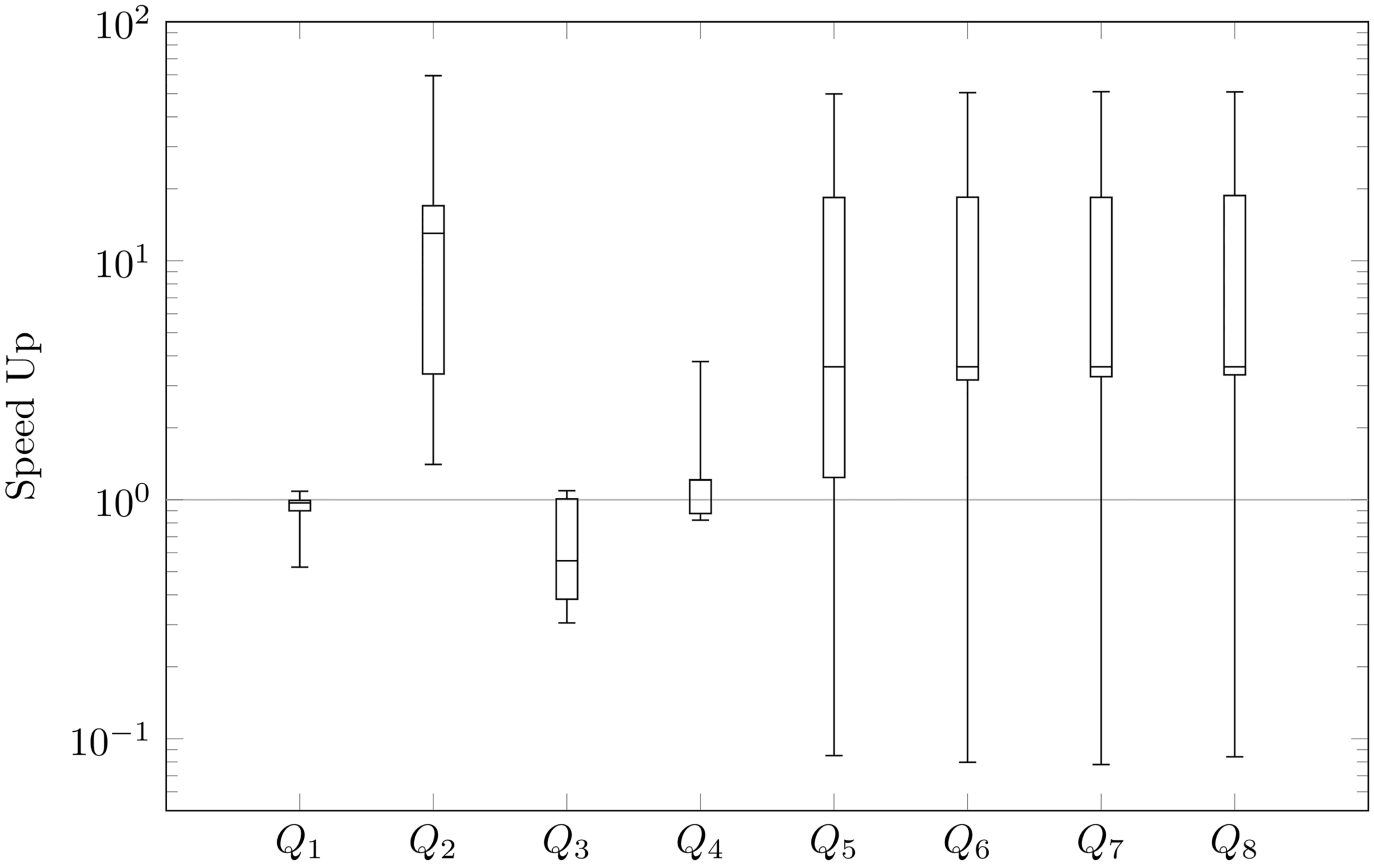
Speed-up of OrientDB SQL querying.

**Fig 12:**
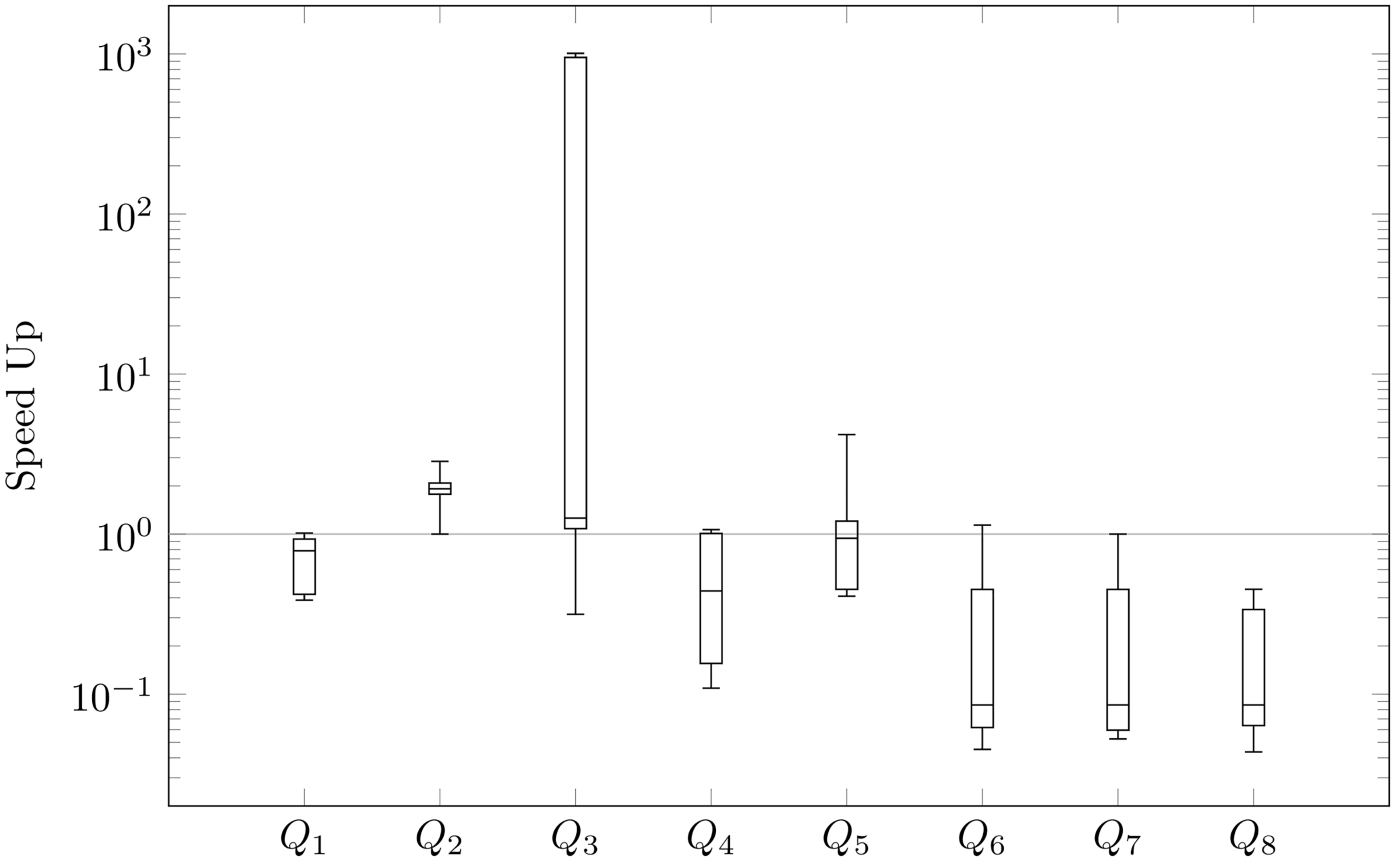
Speed-up of OrientDB Gremlin querying.

We focus mainly on the speed-up of querying, i.e., the ratio of time before to after our reducing for every query: *Q*_1_, *Q*_2_, …, *Q*_8_. In Figs 9, 10, 11 and 12, we present queries in three different databases. OrientDB has been tested using Gremlin [37] and SQL via a console. In Neo4j, we used Cypher Query Language [38], and, in Titan, we executed Gremlin queries. As we expected to reduce the number of vertices, we generally sped-up the process of querying. Indeed, the average amount of speed-up is 24.99 times. However, the speed-up is significantly dependent on the choice of a graph database. This parameter is 2.73 in Neo4j, 38.78 in Titan, 48.52 in OrientDB via Gremlin, and 9.92 in OrientDB via a console. Based on OrientDB via Gremlin we obtain the biggest average number of speed-up, however the ratio of time is greater that 1 only in 35% of querying. Note for comparison that Neo4j in 75%, OrientDB via a console in 78.7%, Titan in 82.5%, obtains speed-up greater that 1. If we compare the ratio of time, we can also compare the ratio of time in different graph databases for a particular query. Titan in 52.5%, OrientDB via a console in 25%, Neo4j in 25%, OrientDB via Gremlin in 7.5% of questions obtains the maximum speed-up based on our modification of property graphs. Additionally, OrientDB via Gremlin in 45%, OrientDB via console in 22.5%, Neo4j in 22.5%, Titan in 10% obtain the minimum speed-up.

Our queries can be divided into two types. The first one checks basic operations (loading data—*Q*_1_, select all nodes with properties—*Q*_2_, select all the edges with all properties—*Q*_3_, removing data—*Q*_4_). The second one checks finding the shortest path (*Q*_5_—max depth 2, …*Q*_8_—max depth 5). The analysis showed that our reduction of the number of vertices has different effects in each individual graph database that we considered. Indeed, the average speed-up of querying obtain 96.51 times in the case of the first type of queries in OrientDB via Gremlin, even if the average speed-up in the case of the second type of queries in the graph database is 0.53, i.e., the shortest paths are found about two-times slower after the modification than prior to it. However, such situations occur only in the case of OrientDB via Gremlin. The best results are obtained in the case of Titan, where the average speed-up is 30.90 times in the case of the first type of queries and 46.67 in the second one.

### 4.4 Serializations

In this subsection, we compare efficiency of our reducing vertices in common graph serialization formats, such as GML [39], GraphML [40], and GEXF (https://gephi.org/gexf/format/). This is important because format affects the effective property graph data storage and transmission. This feature is useful for saving disk space, better use of database memory buffers or faster communication between a database client and a server.

Table 5 shows distance-1 characteristics of serializations before and after our vertices reduction. All results are better after our transformation. The best ratio belongs to GML (ds_1_, ds_2_, ds_4_ and ds_5_) and GEXF (ds_3_).

**Table 5:** Distance-1 characteristics of serializations.

Format	Before [*B*]	After [*B*]	Ratio [%]
ds_1_			
GEXF	2234355	17154	130.25
GML	2247187	14369	156.39
GraphML	3233759	2385431	1.36
ds_2_			
GEXF	751755	11718	64.15
GML	832062	6789	122.56
GraphML	1177919	795143	1.48
ds_3_			
GEXF	115815	7432	15.58
GML	116634	8108	14.39
GraphML	150243	139259	1.08
ds_4_			
GEXF	684894	57395	11.93
GML	679809	48539	14.01
GraphML	911459	835754	1.09
ds_5_			
GEXF	928576	8257	112.46
GML	1029485	9008	114.29
GraphML	256936	154732	1.66

In Table 6, we introduce distance-2 characteristics of serializations before and after our vertices reduction. Only two cases have slightly worse results (GML in ds_1_ and ds_3_). It is evident that additional information about the original graph (especially information about the edges) has a great impact in case of a small reduction of nodes. The rest of the serializations give slightly better results than before our transformations.

**Table 6:** Distance-2 characteristics of serializations.

Format	Before [*B*]	After [*B*]	Ratio [%]
ds_1_			
GEXF	466185	462825	1.01
GML	470181	521293	0.90
GraphML	650897	541243	1.20
ds_2_			
GEXF	4730709	1391227	3.40
GML	4769025	1344635	3.55
GraphML	6466636	5007073	1.29
ds_3_			
GEXF	559304	553351	1.01
GML	564097	637539	0.88
GraphML	781198	637506	1.23
ds_4_			
GEXF	29082	9872	2.95
GML	29331	10519	2.79
GraphML	40604	24764	1.64
ds_5_			
GEXF	306051	130496	2.35
GML	318587	117081	2.72
GraphML	503349	292093	1.72

## 5 Related work

### 5.1 Property graphs

In this section, we discuss property graph approaches and solutions. They can be divided into four groups: abstraction layer and formalization of property graphs, property graphs databases, multi-modal databases that support property graphs, and distributed processing frameworks.

The first group relates to proposals that formalize property graphs [29, 41–43]. In [41], Hartig proposes a formalization of the PG model and introduces transformations between PGs and RDF* [44]. Unfortunately, this model is not widely supported by graph stores. In [29], Jouili *et al*. suggested another definition of PG based on Blueprints (https://github.com/tinkerpop/blueprints/wiki). The PG definition is restrictive, because it assumes that labels must be unique. In this paper, authors present a distributed graph database comparison framework. In [42], Schätzle *et al*. present a formalization of PG in the RDF context. Moreover, the paper introduces a SPARQL query processor for Hadoop called S2X. Unfortunately, this paper focuses on distributed storage and do not formalize property graphs in the graph database context. In [43], Batarfi *et al*. propose a formalization of attributedgraphs, which is similar to PGs.

There are a few data stores in the Property Graph world [30, 45]. Neo4j [30] is a native graph database purpose-built to leverage not only data but also its relationships. Titan [29] is another graph database that is distributed and transactional. Dex/Sparksee [45] is yet another graph database, which supports data constraints to guarantee the integrity of data and relationships among them.

The third group is a multi-modal database that supports property graphs, and it can be divided into two subgroups: graph databases that support RDF and PG models [46, 47], and databases that support documents and graphs [31, 48]. Oracle database with Oracle Spatial and Graph option [46] is an add-on database feature with advanced spatial capabilities enabling the development of complex geographic information systems. Bigdata/Blazegraph [47] is another graph database that supports RDF and PG models. It is an ultra-scalable and high-performance database that supports up to 50 ⋅ 10^9^ edges on a single machine. The next subgroup is OrientDB [31] and AranoDB [48]. The first solution supports different indexes and ACID transactions guaranteeing that all database transactions are processed reliably. In AranoDB documents are grouped into collections, that can be related to vertices or edges.

The last group is distributed processing frameworks that use property graphs [49, 50]. GraphX [49], which is an API of Apache Spark for graphs and graph-parallel computation, is an example of property graph usage. It extends the Spark RDD abstraction by introducing the resilient distributed Property Graph. SGraph, a part of GraphLab [50], is another scalable graph data structure, that derives from the property graph idea.

### 5.2 Graph coloring

Graph coloring is known to be an NP-complete problem from the 70s [19, 51, 52]. Since then, the difficulty of this problem has contributed to proposed heuristic algorithms, hoping that the number of colors they use is near optimal [53–55]. Recently, the problem of minimum graph coloring is being better acquainted. Bellare *et al*. [56] show that minimum graph coloring cannot be approximated better than |*V*|^1/7−*ϵ*^ for every *ϵ* > 0, unless *P* = *NP*. Moreover, Feige *et al*. [57] prove that if NP-problems cannot be solved by a randomized algorithm in polynomial time, the minimum graph coloring cannot be approximated better than *Ω*(|*V*^1−*ϵ*^|).

On the other hand, there are numerous heuristic algorithms for specialized graph coloring problems [58–61]. Qu *et al*. [58] propose a hybrid heuristic approach based on estimation distribution algorithms. This paper provides solution of acceptable quality for a number of optimisation problems and demonstrates the generality through experimental results for different variants of exam timetabling problems. FOO-PARTIALCOL is another approach presented in [59]. This method is based on tabu search [62]. A solution consists of *k* disjoint stable sets and a set of uncolored vertices. Yet another approaches are introduced in [60], which are based on Greedy algorithm [63]. In the first paper, Iterated Greedy algorithm is effective in graphs with *n* vertices partitioned into *k* as nearly equal sized sets as possible. In the second paper, the authors show heuristic methods to color vertices of a graph, which relies upon the comparison of the degrees and structures of graphs.

Several coloring algorithms occur in this context, depending on whether the matrix is a Jacobian [64, 65] or a Hessian [66, 67]. In [64], authors apply a column intersection graph-based formulation. In [65], authors propose formulation which is based on the concept of a consistent row-column partition, in which the entire set of rows and columns is partitioned into two respective sets of groups. Vertices that remain uncolored at the end of the algorithm form an independent set in the graph and can be assigned a neutral color zero. Coleman *et al*. [66] propose a model that exploits symmetry. This model is called *path coloring* and requires that every pair of adjacent vertices get distinct colors, and every path on four vertices uses at least three colors. McCormick [67] introduces a graph coloring model for the computation. The model uses the adjacency graph representation of the underlying symmetric matrix and requires that in every path *u*, *v*, *w* in the graph, vertices *u*, *v*, and *w* receive distinct colors.

### 5.3 Duplicate detection and deduplication

There are numerous approaches to duplication and similarity detection in general [68–76]. We distinguish probabilistic approaches [68, 69], supervised machine learning approaches [71, 72, 75] and unsupervised machine learning approaches [70, 73, 74, 76]. There are also solutions for duplication and similarity detection in graph data [77–80]. Dong *et al*. [77] present duplicate detection in a scenario where relationships between publications, persons, etc., form a graph. At each iteration, the first pair in the priority queue is retrieved, compared, and classified as nonduplicate or duplicate. The presented algorithm gradually enriches references by merging attribute values. Kalashnikov *et al*. [78] propose a domain-independent data cleaning approach for a graph of entities. Presented algorithm uses clustering techiques. Yin *et al*. [79] present linkage-based clustering, in which the similarity between two objects is measured based on the similarities between the objects linked to them. Bhattachary *et al*. [80] propose another algorithm that evaluates similarities of candidate pairs at each iterative step, and selects the most similar pair at each iteration. An algorithm augments a general class of attribute similarity measures with relational similarity among the entities. Duplicates are merged together before the next iteration, so that in effect clusters of candidates are compared. This merge updates the reference graph and the priority queue.

On the other hand, we distinguish among approaches for deduplication: active learning methods [81, 82], clustering methods [83], and graph algorithms [84]. Sarawagi *et al*. [81] show how machine learning techiques could be applied in the elimination of redundant data where training data were available. Georgescu *et al*. [82] propose approach for deduplication with the main advantage of using crowdsourcing as a training and feedback mechanism. Culotta *et al*. [83] propose a conditional random field model of duplicate removal that captures these relational dependencies, and then employ a relational partitioning algorithm to jointly deduplicate data. Zhou *et al*. [84] show how to reduce the number of slow synchronization operations needed in parallel graph search.

## 6 Conclusions and future work

Reducing vertices is an important issue in graph databases. In this article, we outlined a way of reducing nodes of a property graph with the use of a graph vertex coloring method. We also presented works from the property graph coloring research area. Finally, we presented experiments, that showed great potential for the presented approaches.

The proposed approach is destined to evolve and include a wider set of coloring methods in its future versions. We showed that when reducing the number of vertices with one property, we can significantly increase the efficiency of working with graph databases that store RDF data, where the average number of speed-up is 24.99 times. However, the speed-up is significantly dependent on the choice of a graph database and a coloring method. Our initial research showed that we obtain large differences in the speed-up, even if we only focus on a single request in different graph databases.

Therefore, as part of future work, we will consider various algorithms for coloring graphs to find the ones that guarantee the best acceleration in the known graph databases. Furthermore, we will try to indicate which ones are best suited to each database. These studies will require not only coloring algorithms with the best approximation, but also heuristics that do not have constant approximations. Moreover, future work will focus on property graph partitions.
